# The Microbiome and Esophageal Disease: Where Are We Now?

**DOI:** 10.1007/s11894-026-01044-3

**Published:** 2026-05-14

**Authors:** Ping He, Ellen Stein

**Affiliations:** 1 Division of Gastroenterology and Hepatology, Robert Wood Johnson School of Medicine, Rutgers University, New Brunswick, NJ USA; 2 Robert Wood Johnson School of Medicine, Rutgers University, New Brunswick, NJ USA

**Keywords:** Microbiome, Esophageal diseases, Microorganisms, Esophageal microbiome, Next generation sequencing

## Abstract

**Purpose of Review:**

The microbiome in the esophagus has been an area of early active investigation. In the past 5 years, advancements in next-generation sequencing and computational analysis have provided a more detailed picture of the esophageal microbiome. This review will examine the most recent advances in the past 5 years on the study of microbiome changes in healthy and diseased esophagus to better understand the link between microbiome and esophageal diseases, potentially leading to new prevention or treatment strategies.

**Recent Findings:**

The microbiome has a key set of baseline parameters in the usual or non-diseased esophageal lumen. There are measurable changes in the baseline microbiome species of different disease phenotypes including eosinophilic esophagitis, gastroesophageal reflux disease, Barrett’s esophagus and achalasia. Some studies suggest that certain microbiome features may even be associated with worse outcomes.

**Summary:**

Dysbiosis of esophageal microbiome is implicated in various esophageal disorders, including GERD, Barrett’s esophagus, EoE, and achalasia. However, confounding factors such as antibiotic use, PPI use, dietary habits, and geographic location influence microbiome variability and make the standardized study of this field challenging. The next phase of research will need to include more focus on larger scale studies with reproducible parameters. As some features of the microbiome are associated with disease progression, there are multiple new avenues of intervention potentially available with an improved understanding of the human esophageal microbiome.

## Introduction

While the importance of microorganisms in human diseases is increasingly being recognized, the esophageal microbiome remains a mystery. Recent advancements in technology have revealed distinct microflora profiles associated with various esophageal diseases like gastroesophageal reflux disease (GERD), Barrett’s esophagus, eosinophilic esophagitis (EoE), and esophageal carcinoma. This review will examine the most recent advances in the past 5 years on the study of microbiome changes in healthy and diseased esophagi to better understand the link between microbiome and esophageal diseases, potentially leading to new prevention or treatment strategies.

### Characterizing the Healthy Esophageal Microbiome

Earliest attempts in the 1980’s to characterize the esophageal microbiome using traditional isolation and culture disproved the accepted notion at the time that the esophagus is a sterile space [1, 2]. *Streptococcus* was identified as the most common species in the esophagus [3]. However, those early studies underestimated the complexity of the esophageal microbiome due to limitations in sample collection, culture, and identification. The current gold standard for identification and study of microbiome using 16S ribosomal RNA sequencing became more available in the early 2000s. This method was used to confirm the prevalence of Firmicutes (especially *Streptococcus*) which along with five other dominant flora comprise the majority of the normal esophageal microbiome [4]. While research on the healthy esophageal microbiome was still limited before 2020, it was known that gram-positive bacteria, particularly *Streptococcus*, are the predominant microorganisms in the esophagus alongside other phyla like Proteobacteria, Bacteroidetes, and Actinobacteria [5, 6].

Recent advancements in next-generation sequencing and computational analysis have provided a more detailed picture of the esophageal microbiome. Table 1 summarizes the studies of the esophageal microbiome in various diseases. In 2023, Gilroy et al., leveraging both DNA sequencing and metagenomic analysis of esophageal brushings from UK and Australian cohorts, established a high-resolution, species-level database, identifying 63 bacterial species, including novel and cultured species [7]. It highlighted the significant variability in esophageal microbiome composition, with no single species present in all samples and only 50 species shared between the cohorts [7]. The strong correlation between salivary and esophageal samples within the same individual, compared to samples of the same type across individuals, underscores the substantial influence of the oral microbiome on the esophagus [7, 8]. A 2024 population-based study of more than 200 participants examined the connection between salivary microbiome and Upper GI Disorders [9]. They demonstrated that saliva dysbiosis was linked to several UGI conditions, including GERD, symptomatic esophagitis, as well as combined esophagitis and Barrett’s esophagus [9]. In 2024, She et al. expanded the understanding of the microbiome across different organs in the same individual by profiling the microbiome across seven surface organs of deceased individuals, revealing that the esophagus exhibits significantly higher α-diversity than other gastrointestinal regions [10]. Specific phyla enriched the proximal and distal portions of the esophagus and stomach, with Proteobacteria and Firmicutes dominating the esophagus and stomach, and Helicobacter species showing particular enrichment in these upper gastrointestinal tracts, especially the thoracic esophagus [10]. Furthermore, functional analysis indicated an enrichment of bacteria involved in lipid metabolism in the esophagus and stomach [10]. These studies collectively demonstrate that the esophageal microbiome is a unique and diverse ecosystem, heavily influenced by the oral microbiome, with distinct regional and functional characteristics. However, the observed inter-individual and population-based variability emphasizes the limitations of generalizing findings from single-cohort studies and underscores the need for larger, more diverse investigations to fully characterize the esophageal microbiome and its role in health and disease.

**Table 1:** Summary of studies on esophageal microbiome

Study	Country	Population	Analysis	Main findings
Normal Esophageal Microbiome				
Gilroy et al. 2023	United Kingdom, Australia	11 patients in U.K. undergoing endoscopy and publicly available data from 50 patient from Australia	16S rRNA sequencing of oral swabs and biopsy samples	Of 60 species found in at least one sample, no species was present in all samples, and 50 species are found in both cohorts
She et al. 2024	China	33 donors after cardiovascular death in China	16S rRNA sequencing of surface swabs and biopsies	Among upper GI tract (oral cavity, esophagus, stomach), esophagus has the highest α-diversity before it decreases in the stomach. Bacteroidetes, Actinobacteria, and Fusobacteria were enriched in large intestine, skin, and oral cavity, respectively, while Proteobacteria and Firmicutes were enriched in esophagus, stomach, and small intestine
Gastroesophageal Reflux (GERD)/Barrett’s Esophagus (BE)/Esophageal Adenocarcinoma (EAC)				
Zhou et al. 2020	Australia	70 patients in Australia (16 control, 11 non-erosive reflux disease, 20 reflux esophagitis, 17 BE, 6 EAC)	16S rRNA sequencing of biopsies	Reflux esophagitis and Barrett’s esophagus compared to control had an increase in Proteobacteria and Bacteroidetes and a decrease in Fusobacteria and Actinobacteria. In esophageal adenocarcinoma, microbiome is shifted towards Firmicutes and Proteobacteria, while away from Actinobacteria relative to the control
Okereke et al. 2021	United States	74 patients (34 BE, 40 Control)	DNA, microbiome qPCR array	10 different genera (*Corynebacterium*, *Dialister*, *Gemella*, *Haemophilus*, *Leptotrichia*, *Neisseria*, *Prevotella*, *Rothia*, *Streptococcus*, *Veillonella*) are less likely to be detected as length of Barrett’s esophagus increases
Hao et al. 2022	United States	121 patients (27 normal subjects, 37 GERD,32 BE and 25 EAC)	16S rRNA sequencing of biopsies	*Streptococcus* gets progressively depleted during progression of normal esophagus, gastroesophageal reflux, Barrett’s esophagus, and esophageal adenocarcinoma while six other genera *Atopobium*, *Actinomyces*, *Veillonella*, *Ralstonia*, *Burkholderia* and *Lautropia* are progressively enriched
Ridani et al. 2022	Germany	48 patients (15 Barrett’s esophagus, 4 dysplasia, 15 EAC, and 14 healthy) from Munich and 75 frozen biopsy samples from previous study from New York	16S rRNA sequencing of biopsies, saliva, fecal samples	PAX gene is a valid method for microbiome RNA collection. No changes in oral and fecal microbiome between disease states. In Munich group, Decrease Actinobacteria in EAC compared with all other groups and an increase of the phylum of Firmicutes in EAC compared with BE. In NY group, Bacteroidetes decrease in EAC and dysplasia compared with healthy, and Fusobacteria a decrease in EAC compared with healthy
Zaramella et al. 2023	Italy	80 patients (57 non-dysplastic BE, 8 low-grade dysplastic BE, 8 high-grade dysplasia, and 7 EAC)	16S rRNA sequencing of biopsies	Bacteroidetes was higher in adenocarcinoma group compared to control but the difference was not statistically significant. *Patescibacteria* is more abundant for low-grade dysplasia compared to BE without dysplasia
Power et al. 2024	Australia	58 patients (25 normal and 33 with reflux disease based on DeMeester score)	16S rRNA sequencing of mouthwash samples	Species of Firmicutes, Bacteroidetes, and Spirochaetes were more abundant in the reflux groups than in the normal group, while Proteobacteria is more abundant in the normal group compared to reflux group
Solfisburg et al. 2024	United States	244 patients (125 non-BE, 119 BE)	16S rRNA sequencing of saliva samples	*Streptococcus* increased in oral microbiome dysbiosis associated with HGD/EAC, independent of tooth loss. Metabolic modeling predicts increases in L-lactic acid and decreases in butyric acid and L-tryptophan production in HGD/EAC
Sadhegi et al. 2024	Sweden	388 adults underwent upper endoscopy (microbiota in 380 saliva, 200 subgingival, and 267 buccal mucosa samples collected during endoscopy)	16S rRNA sequencing of oral samples	Increase in *Atopium* in esophagitis and Barrett’s esophagus. 10 times enrichment of *Fretibacterium* and in buccal mucosa, 3 times enrichment of *Fusobacterium* were noted in the Barrett’s esophagus
Zhang et al. 2024	United States	24 children younger than 18 years old	16S rRNA sequencing of stool and oropharyngeal samples before and after 8 weeks of PPI	*Streptococcus* increased, whereas *Bifidobacterium*, *Peptostreptococcaceae*, and *Turicibacter* all decreased in abundance after PPI use
Tanes et al. 2025	United States	153 patients (52 controls, 101 BE: 50 no dysplasia, 10 indefinite, 17 low-grade dysplasia, 17 high-grade dysplasia, and 7 EAC)	16S rRNA sequencing of saliva, oral rinse, esophageal squamous brushings, and BE	*Streptococcus* increased in BE in both squamous and Barrett’s brushings. Conversely, *Prevotella, Alloprevotella* and *Fusobacterium* decreased in BE patients in both squamous and Barrett’s brushings and *Actinomyces* decreased only in Barrett’s brushings With progression from BE to EAC, *Lactobacillus* increase with progression. *Actinomyces*, *Prevotella*, *Neisseria, Fusobacterium, Megasphaera, Selenomonas*, and *Oribacterium* decrease with progression toward EAC
Eosinophilic Esophagitis (EoE)				
Johnson et al 2021	United States	24 EoE patients and 25 control patients	16S rRNA sequencing of biopsy samples	No microbiome differences between EoE and Control samples and no differences within EoE group associated with clinical phenotype, presence of atopy, or endoscopic features
Laserna-Mendieta et al. 2021	Spain	30 EoE patients (active at baseline, remission after PPI (n = 10), topical steroid (n = 10), food elimination (n = 10)) and 10 controls	16S rRNA sequencing of biopsy samples	No difference in α-diversity of EoE compared to control. Patients undergone food elimination had lower microbial diversity. Microbiome after steroid treatment is more similar to that of control. Overall, no major difference in the composition of the microbiome between groups. *Filifactor*, *Parvimonas* and *Porphyromonas* genera were less abundant in baseline samples from EoE patients. *Filifactor* and *Porphyromonas* decrease after treatment, while *Parvimonas* show recovery
Furuta et al. 2022	United States	93 EoE, 17 EoG, and 29 control specimens (18 esophageal) from 10 sites across the United States	16S rRNA sequencing of biopsy samples	*Streptococcus* is reduced in active EoE. *Streptococcaceae* increased primarily in inactive EoE. *Gemella* and *Actinobacillus* increased in EoE irrespective of disease status
Massimino et al. 2023	Italy	38 controls, 43 EoE, 10 GERD across 18 different studies	16S rRNA sequencing of biopsy samples	*Streptococcus mitis* and *Hemophilus parainfluenzae* are associated with EoE. Bacterial species diversity is higher in EoE compared to control
Sasahira et al. 2025	Japan	59 patients were enrolled (8 inactive EoE, 17 active EoE, and 28 non-EoE controls)	16S rRNA sequencing of saliva and mucosal biopsy samples	In mucosal samples, *Prevotella* increased in EoE and *Streptococcus* decreased In saliva samples, *Prevotella* is higher in active EoE and *Neisseria* are lower in active EoE
Achalasia/Esophageal Dysmotility				
Takahashi et al. 2021	Japan	6 patients with achalasia and 14 with superficial ESCC	16S rRNA sequencing of biopsy samples	Relative abundance of *Haemophilus* and *Neisseria* increased in the esophagus after POEM
Jung et al. 2022	South Korea	Samples before and after POEM from 29 patients across 4 centers in South Korea	16S rRNA sequencing of biopsy samples	Esophageal microbial composition showed no significant changes 8 weeks post POEM
Yeh et al. 2023	Taiwan	31 achalasia patients and 15 controls	16S rRNA sequencing of biopsy samples	Increased Firmicutes and decreased Proteobacteria comparing achalasia to control. *Lactobacillus*, *Megasphaera* and *Bacteroides* are enriched in achalasia patients and the amount of Lactobacillus was associated with the severity of achalasia. After POEM, there is an increase in *Neisseria* and decrease in *Lactobacillus* and *Bacteroides*

Abbreviations: *HGD* High-grade Dysplasia; *EoG* Eosinophilic Gastroenteritis; *ESCC* Esophageal Squamous Cell Carcinoma; *POEM* Peroral Endoscopic Myotomy

### Effects of Microbiome on GERD and Barrett’s Esophagus

Since the late 2000 s, research on the esophageal microbiome increasingly explored its role in gastroesophageal reflux disease (GERD) and Barrett’s esophagus (BE), conditions characterized by acid reflux, heartburn, and potential progression to esophageal adenocarcinoma. While traditional understanding focused on acid-induced inflammation, early studies of the microbiome suggested that GERD and BE change the esophageal microbiome, particularly leading to an increase in gram-negative bacteria like *Fusobacterium*, *Neisseria*, and *Veillonella*, and a decrease in *Streptococcus* [5, 6]. These microbial shifts, including variations in phyla like Firmicutes, Proteobacteria, and Bacteroidetes, were hypothesized to contribute to the pathogenesis of GERD and BE and offer potential biomarkers for predicting BE progression [11, 12]. Most of the earlier studies have shown differences in the esophageal microbiome in GERD and BE, but some did not and this discrepancy could be attributed to small sample size of the studies, different study populations, proton pump inhibitor use, and different methods of sample collection and microorganism identification [13].

Research exploring the impact of (GERD) and proton pump inhibitors (PPIs) on the oral and esophageal microbiome remains under investigation. In a pediatric study of stool and oropharyngeal samples from 20 patients, PPI use was associated with an observed enrichment of *Streptococcus* in the stool after PPI use and a reduction in the relative abundance of *Bifidobacterium*, *Peptostreptococcus*, and *Turicibacter* [14].In GERD patients, varying degrees of reflux severity correlate with specific alterations in oral microbiota, including reductions in *Rothia* species and increases in other bacterial genus [15]. PPI use in adults does not appear to significantly alter the distal esophageal microbial community, except for only minor changes in *Actinomyces* levels, regardless of PPI dose or duration [16]. In contrast, in children with a normal esophagus, PPI exposure is associated with upregulation of genes involved in esophageal mucosal homeostasis and epithelial cell function, along with changes in the active esophageal microbiota, notably an increased abundance of *Prevotella* and *Streptococcus* [17]. These findings provide limited evidence suggesting that GERD severity and PPI use, particularly in children, can influence microbial communities and gene expression in the upper gastrointestinal tract.

There is great interest in preventing the progression from reflux to cancer and several studies of the esophageal microbiome have focused on the progression of GERD to BE to esophageal adenocarcinoma (EA). Table 2 offers a look at studies focusing on changes in the microbiome in esophageal disease states. Studies using endoscopic biopsy samples from all three stages of pre-oncogenic to oncogenic progress have shown significant shift in the microbiome of the oral cavity and esophagus. Specifically, antibiotic use may contribute to this progression by altering microbial diversity, reducing beneficial *Streptococcus* populations, and enriching procarcinogenic bacteria like *Atopobium*, *Actinomyces*, and *Veillonella*, alongside increased antibiotic resistance genes and pathways related to inflammation and carcinogenesis [18]. Researchers have since begun to account for antibiotic use within 3 months of study assessment to avoid false measurement. This dysbiosis, characterized by a simplified microbial network and altered gene functionality, is observed along the GERD-BE-EA progression [18]. However, a later study by Zaramella et al. did not show the same decrease in Firmicutes and the diversity of the microbiome previously described [19]. Instead, a significant increase in the Gram-negative order Bacteroidales (Bacteroidetes phylum) was observed along the disease progression [19]. The inter-study inconsistencies between microbiome changes during progression may be due to the alpha diversity in microbiomes of healthy esophagi among different study populations. This again highlights the importance of studies with large sample sizes across different populations to identify more generalizable patterns of dysbiosis that are associated with disease progression. One study that tried to address the interpopulation differences from Radani et al. collected saliva, fecal and biopsy microbiome samples in a Munich cohort and compared the fecal samples to those from a larger study done in Germany and biopsy samples to those collected from prior studies in New York. They showed that changes in biopsy microbiome samples associated with GERD-BE-EA progression are not reflected in saliva and fecal samples [20]. Furthermore, when comparing the Munich cohort to the New York cohort, although the results between cohorts are not identical, they both had a decline in community diversity with disease progression (alpha-diversity) and distinct microbial profiles between phenotype (beta-diversity) [20]. Analysis of the esophageal microbiome, salivary microbiome, and metabolite modeling has been used to identify microbial signatures associated with different stages of BE and EA, potentially leading to the development of risk prediction models for EA onset for early detection and patient management [19, 21]. The Resident Esophageal Microbiota Dysbiosis Test (REM-DT) proposed by Zaramella et al. uses 6 different risk conditions to assess overall risk of progression to cancer and has a negative predicted value of 96.9% and an accuracy of 85.0% based on their single centered study [19]. The prediction models, microbiome changes and metabolic capacity differences remain to be validated in larger studies before prime-time clinical use for predicting risk of malignant transformation can be entertained. A more robust and better-powered study by Tanes et al. showed that bile acids influence the microbiome in a series of over 100 Barrett’s patients compared to 50 controls. Using 16S rRNA gene sequencing of the V1-V2 hypervariable regions and controlling for PPI use, investigators identified higher levels of Streptococcus appearing in BE patients. Prevotella, Alloprevotella and Fusobacterium were found in lesser frequency. Lactobacillus seemed to be associated with a progressive stage of neoplasia [22].

**Table 2:** Changes in esophageal microbiome in esophageal diseases

Disease	Increase	Decrease
GERD	Proteobacteria and Bacteroidetes (Zhou 2020) *Atopobium*, *Actinomyces*, *Veillonella*, *Ralstonia*, *Burkholderia* and *Lautropia* (Hao 2022) Firmicutes, Bacteroidetes, and Spirochaetes (Power 2024)	Fusobacteria and Actinobacteria (Zhou 2020) *Streptococcus* (Hao 2022) Proteobacteria (Power 2024)
PPI Use	*Streptococcus* (Zhang 2024)	*Bifidobacterium*, *Peptostreptococcaceae*, and *Turicibacter* (Zhang 2024)
Barrett’s Esophagus	Proteobacteria and Bacteroidetes (Zhou 2020) *Atopobium*, *Actinomyces*, *Veillonella*, *Ralstonia*, *Burkholderia* and *Lautropia,* (Hao 2022,) *Atropium, Fretibacterium,* and *Fusobacterium* (Sadhegi 2024) *Patescibacteria* [associated with dysplasia] (Zaramella 2023) *Streptococcus* (Tanes 2025)	Fusobacteria and Actinobacteria (Zhou 2020) *Corynebacterium*, *Dialister*, *Gemella*, *Haemophilus*, *Leptotrichia*, *Neisseria*, *Prevotella*, *Rothia*, *Streptococcus*, *Veillonella* (Okereke 2021) *Streptococcus* (Hao 2022) *Prevotella, Alloprevotella* and *Fusobacterium* (Tanes 2025)
Esophageal Adenocarcinoma	Firmicutes and Proteobacteria (Zhou 2020) *Atopobium*, *Actinomyces*, *Veillonella*, *Ralstonia*, *Burkholderia* and *Lautropia* (Hao 2022) Firmicutes (Ridani 2022) Bacteroidetes (Zaramella 2023) *Lactobacillus* (Tanes 2025)	Actinobacteria (Zhou 2020) *Streptococcus* (Hao 2022) Actinobacteria, Bacteroidetes, Fusobacteria (Ridani 2022) *Actinomyces*, *Prevotella*, *Neisseria, Fusobacterium, Megasphaera, Selenomonas*, and *Oribacterium* (Tanes 2025)
Eosinophilic Esophagitis	*Gemella* and *Actinobacillus* (Furuta 2022) *Streptococcus mitis* and *Hemophilus parainfluenzae* (Massimino 2023) *Prevotella* (Sasahira 2025)	*Filifactor*, *Parvimonas* and *Porphyromonas* (Laserna-Mendieta 2021) *Streptococcus* [active EoE] (Furuta 2022) *Streptococcus* (Sasahira 2025)
Achalasia	Firmicutes; *Lactobacillus*, *Megasphaera* and *Bacteroides* (Yeh 2023)	*Haemophilus* and *Neisseria* [increased post POEM] (Takahashi et al. 2021) Proteobacteria, *Neisseria* [increased post POEM] (Yeh 2023)

Green: Congruent changes between studies. Red: Conflicting changes between studies

### Contribution of Microbiome to Eosinophilic Esophagitis

Eosinophilic esophagitis (EoE) is a chronic, immune-mediated esophageal disease characterized by eosinophilic infiltration, leading to clinical manifestations such as dysphagia, food impaction, and reflux-like symptoms. The pathogenesis of EoE is hypothesized to involve dysbiosis of the esophageal microbiota, influenced by environmental exposures and dietary antigens. Older studies investigating the esophageal microbiome in EoE have yielded variable results. Earlier studies by Harris et al. observed a shift from gram-positive (Firmicutes) to gram-negative (Proteobacteria) dominance in untreated EoE, with a significant increase in *Haemophilus* species [23]. In contrast, Johnson et al. reported no significant differences in microbial diversity between EoE and non-EoE adult cohorts, highlighting the heterogeneity of findings [24]. Benitez et al. demonstrated increased Proteobacteria abundance (specifically *Corynebacterium* and *Neisseria*) in pediatric EoE, with dietary allergen intake correlating with increased *Granulicatella* and *Campylobacter* [25]. Norder Grusell et al. found increased microbial diversity in EoE compared to GERD and healthy controls, with *Streptococcus viridans* being the most prevalent bacteria and increased *Haemophilus* and *Prevotella* abundance in EoE [26]. Laserna-Mendieta et al. documented Firmicutes as the dominant phylum in both EoE and non-EoE, with a slight increase in Bacteroidetes and decrease in Proteobacteria in active EoE [27]. These studies collectively suggest a potential role for microbial dysbiosis in EoE pathogenesis, though the precise nature and clinical significance of these alterations remain unknown.

In more recent years, standardized multi-cohort studies have helped to further define the specific microbial signatures associated with EoE and to explore their potential as diagnostic or therapeutic targets. In the largest study so far, Furuta et al. investigated the mucosal microbiota composition in mucosal biopsies from patients with eosinophilic esophagitis (EoE) and eosinophilic gastritis (EoG) patients using a cohort of 93 EoE, 17 EoG, and 29 control specimens (18 esophageal) from 10 sites across the United States [28]. Results revealed significant differences in dominant bacterial taxa between Eosinophilic Gastrointestinal Diseases (EGIDs) and controls, with increased Streptococcus in the esophagus of EoE patients and Prevotella in the stomach of EoG patients [28]. Specific taxa, including Streptococcus and Gemella for EoE, and Leptotrichia for EoG, were associated with active disease [28]. Like previous mentioned studies, high inter-individual variability was observed, and stool microbiota did not correlate with mucosal findings [28]. Alpha diversity metrics were similar across groups [28]. More recent study of 59 patients by Sahahira et al. showed that Prevotella was significantly more abundant in the inactive EoE group than in the control group, and it was able to be measured in the active EoE group as well. *Streptococcus*, the most common genus of the esophagus, was significantly less common among those with active EoE disease, compared to those with inactive or no EoE. Endoscopic findings of EoE were inversely correlated with *Streptococcus*, showing some propensity for more furrows and edema in those with less Streptococcus. This may suggest further study into a probiotic with Streptococcus to help with EoE is possible [29]. A bioinformatics approach has also been used to study the microbiome difference in EoE and to overcome the high variability between individuals and lack of larger prospective studies. Massimino et al. performed multi-omic meta-analysis on transcriptomic and meta-transcriptomic data from 660 human esophageal mucosal sample and blood samples from 18 different studies [30]. Comparison of microbiome profiling within esophageal tissues of patients with eosinophilic esophagitis (EoE), gastroesophageal reflux disease (GERD), and healthy controls revealed that bacterial species were the most significantly dysregulated microbial entities in EoE [30]. *Streptococcus mitis* and *Hemophilus parainfluenzae*, typically found in the oropharynx, are the most abundant among nine candidate species specifically characterizing the EoE esophagus as described by previous studies using small sample sizes [23, 26, 30]. Contrary to some previous studies, Massimino et al. found increased bacterial diversity, but not species dominance, in EoE esophagus compared to controls, possibly due to the larger sample size and increased statistical power [30].

#### Microbiome and Diseases of Esophageal Motility

The role of microbiome alterations in esophageal motility disorders and post-intervention microbial dynamics is an emerging area of research. Chronic esophageal stasis in achalasia induces epithelial inflammation and is associated with increased esophageal squamous cell carcinoma (ESCC) risk; however, the precise relationship between the esophageal microbiome, stasis, and achalasia remains incompletely elucidated. Early study using subculture techniques identified a predominance of aerobic gram-positive bacteria, particularly *Streptococcus*, and anaerobic bacteria, notably *Veillonella*, in megaesophagus compared to controls [31].

Takahashi et al. (2021) conducted a single-center, prospective, observational study in Japan, examining the esophageal microbiome in achalasia patients undergoing peroral endoscopic myotomy (POEM) and ESCC patients undergoing endoscopic submucosal dissection (ESD) [32]. No significant differences in the esophageal microbiome between ESCC and achalasia was found [32]. However, there was a significant increase in *Haemophilus* and *Neisseria* in the esophagus and a decrease in *Fusobacterium* in the oral microbiota post-POEM [32]. Conversely, Jung et al., in a multi-center observational study of 29 achalasia patients pre- and post-POEM, found no significant microbiome changes 8 weeks post-procedure [31]. Both studies confirmed the dominance of Firmicutes, Bacteroidetes, Proteobacteria, Actinobacteria, and Fusobacteria, with *Streptococcus* being the most abundant genus. Discrepancies in post-POEM microbiome changes may stem from small sample sizes or post-procedural acid reflux. Notably, neither study included a control group.

Yeh et al. conducted a 16S rRNA sequencing-based case–control study, enrolling achalasia patients and asymptomatic controls [33]. Esophageal brushing specimens were collected, with follow-up endoscopy and brushing performed 3 months post-POEM [33]. Achalasia patients (n = 31) exhibited a distinct microbial community structure, characterized by increased Firmicutes and decreased Proteobacteria at the phylum level, compared to controls (n = 15) [33]. Enriched genera in achalasia included *Lactobacillus*, *Megasphaera*, and *Bacteroides*, with *Lactobacillus* abundance correlating with achalasia severity [33]. In post-POEM patients (n = 20), a high prevalence of erosive esophagitis (55%) was observed, accompanied by increased *Neisseria* and decreased *Lactobacillus* and *Bacteroides* genera [33].

Ikeda et al. investigated the pathogenesis of achalasia, focusing on esophageal smooth muscle contractility, inflammation, and the role of the esophageal microbiota [34]. Analysis of lower esophageal sphincter (LES) mucosal samples indicated significant hypophosphorylation of myosin light chain 20 (LC20) in the LES of achalasia patients, potentially contributing to impaired contractility, and associated with downregulation of CPI-17 [34]. Furthermore, a pronounced Th17-mediated inflammatory response, characterized by elevated IL-17A, IL-17F, IL-22, and IL-23A, was observed [34]. Alterations in the esophageal microbiota, including increased α-diversity and enrichment of *Actinomyces* and *Dialister*, correlated with Th17 cytokine levels [34]. Transfer of esophageal-conditioned media from achalasia patients to mice induced elevated IL-17F levels [34]. These findings suggest that impaired LES contractility due to LC20 hypophosphorylation, driven by CPI-17 downregulation, and a dysbiotic esophageal microbiome contributing to Th17-mediated inflammation, are key factors in achalasia pathogenesis.

## Conclusion

The esophageal microbiome is a complex and dynamic ecosystem that plays a significant role in both esophageal health and disease. Recent advancements in sequencing technologies have greatly enhanced our understanding of the microbial composition and functional attributes of the esophagus, revealing a diverse esophageal microbiome heavily influenced by the oral microbiome. Dysbiosis of the esophageal microbiome is implicated in various esophageal disorders, including GERD, Barrett’s esophagus, EoE, and achalasia. Characterizing specific microbial signatures associated with these conditions holds promise for the development of novel diagnostic and therapeutic strategies, such as risk prediction models and targeted microbiome interventions.

However, significant inter-individual and inter-study variability in esophageal microbiome composition highlights the need for larger, well-controlled, multi-center studies. These future investigations should account for confounding factors such as antibiotic use, PPI use, dietary habits, and geographic location to validate current findings and elucidate the precise mechanisms by which the microbiome influences esophageal pathophysiology. Given the current paucity of large-scale studies across diverse populations, bioinformatic integration of data from multiple studies offers a promising strategy to enhance standardization and mitigate interstudy variability. A more comprehensive understanding of the interplay between the esophageal microbiome, host genetics, and environmental factors is crucial for translating microbiome research into clinically actionable applications and improving patient outcomes.

## Key References

- She JJ, Liu WX, Ding XM, Guo G, Han J, Shi FY, et al. Defining the biogeographical map and potential bacterial translocation of microbiome in human ‘surface organs’. Nat Commun. 2024;15(1):427.
  - Most extensive microbiome sequencing study done to define the normal microbiome throughout body surfaces including the esophagus.
- Sadeghi F, Sohrabi A, Zagai U, Andreasson A, Vieth M, Talley NJ, et al. Oral Microbiome Dysbiosis Is Associated With Precancerous Lesions and Disorders of Upper Gastrointestinal Tract: A Population-Based Study. Am J Gastroenterol. 2024;120(9):2173–85.
  - Large well-designed study showing association between changes in oral microbiome and upper GI disorders. Specifically, the buccal and subgingival microbiome changes are associated with premalignant conditions such as Barrett’s esophagus and atrophic gastritis
- Tanes C, Li Y, Falk GW, Ginsberg GG, Wang KK, Iyer PG, et al. Increased reflux secondary bile acids are associated with changes to the microbiome and transcriptome in Barrett’s esophagus. Gut Microbes. 2025;17(1):2545420.
  - Detailed multi-center, cross-sectional study examining the influence of bile acids on the microbiome and relationship to Barrett’s esophagus. Bile acids metabolism and their role in microbiome changes in disease are now being more appreciated in various GI microbiomes.
- Sasahira M, Matsumoto H, Go TT, Yo S, Monden S, Ninomiya T, et al. The Relationship Between Bacterial Flora in Saliva and Esophageal Mucus and Endoscopic Severity in Patients with Eosinophilic Esophagitis. Int J Mol Sci. 2025;26(7).
  - Largest study so far showing association between microbiome changes and EoE. Most significantly, this study showed the EREFS of EoE and *Streptococcus* were inversely correlated, reaffirming this relationship found previously.

## Data Availability

No datasets were generated or analysed during the current study.
